# Correction to Williamson (2014)

**DOI:** 10.1037/trm0000013

**Published:** 2014-10-20

In the article “Towards a Theory of Collective Posttraumatic Growth in Rwanda: The Pursuit of Agency and Communion” by Caroline Williamson (*Traumatology*, 2014, Vol. 20, No. 2, pp. 91–102. http://dx.doi.org/10.1037/h0099393), the copyright attribution was incorrect. The copyright is retained by the author.

Likewise, the following text should have appeared in the author note: “This article has been published under the terms of the Creative Commons Attribution License (http://creativecommons.org/licenses/by/3.0/), which permits unrestricted use, distribution, and reproduction in any medium, provided the original author and source are credited. Copyright for this article is retained by the author(s). Author(s) grant(s) the American Psychological Association the exclusive right to publish the article and identify itself as the original publisher.”

All versions of this article have been corrected.

